# The Impact of Post-traumatic Stress of SARS-CoV-2 Affliction on Psychological and Mental Health of Student Survivors: Cross Sectional Study

**DOI:** 10.3389/fpubh.2022.845741

**Published:** 2022-05-09

**Authors:** Snehil Dixit, Alamin Musa, Audrey Borghi Sillva, Ravi Shankar Reddy, Mohammed Abohashrh, Venkata Nagaraj Kakaraparthi, Faisal Asiri, Flavia Rossi Caruso, Shashi Kumar Govindappa, Arif Ahmad Mohammed

**Affiliations:** ^1^Department of Medical Rehabilitation Sciences, College of Applied Medical Sciences, King Khalid University, Abha, Saudi Arabia; ^2^Cardiopulmonary Physiotherapy Laboratory, Physiotherapy Department, Federal University of Sao Carlos, Sao Carlos, Brazil; ^3^Department of Radiological Sciences, College of Applied Medical Sciences, King Khalid University, Abha, Saudi Arabia; ^4^Department of Basic Medical Sciences, College of Applied Medical Sciences, King Khalid University, Abha, Saudi Arabia; ^5^Department of Physiotherapy, College of Applied Medical Sciences, University of Hail, Hail, Saudi Arabia; ^6^Centre of Excellence in Biotechnology Research, King Saud University, Riyadh, Saudi Arabia

**Keywords:** psychological impact, cognitive impact, post-traumatic stress, SARS-CoV-2, post-traumatic stress disorders (PTSD)

## Abstract

**Background:**

COVID-19 survivor's population is often associated with a long term impact on mental and psychological health. Recent included studies have also stated affliction of mental health due to fear of virus and preventive policies among the college students.

**Objectives:**

The research was conducted to find the psychological and mental impacts of SARS-CoV-2 affliction among the students' survivors in the university.

**Methods:**

The study design of the experiment was cross-sectional, sampling technique was non probability and sampling method being applied was convenience sampling. IBM Statistical Package for the Social Sciences version 20 was used for analyses. Descriptive data was examined and results were showed as mean and standard deviations, percentages, frequencies for continuous variables of IES-R scale (Intrusion, Avoidance, and Hyperarousal) using the total sample of *n* = 34.

**Results:**

Out of 34 only 24 student survivors responded to the online survey post COVID-19 recovery, with an overall participation level of 71%. Grading was given for the total IES-R score which was subdivided into a predefined range. Out of 24 participants, 9 (38%) participants showed the symptoms of mild (*n* = 2)–severe (*n* = 7) psychological impacts. On correlation of factors total IES-R score and taste and sense of smell were moderately correlated. The ordinal regression for complete loss of sense of taste and smell was also significant.

**Conclusion:**

The results from IES-R evaluation clearly outlines the presence of psychological sequels post recovery of COVID-19 episodes among the young college survivors. Complete loss of sense of smell and taste may be an indicator of psychological sequelae as compared to reduce sense of smell.

## Graphical Abstract

Psychological, mental health, and academic performance evaluation post COVID-19 infection among students. 
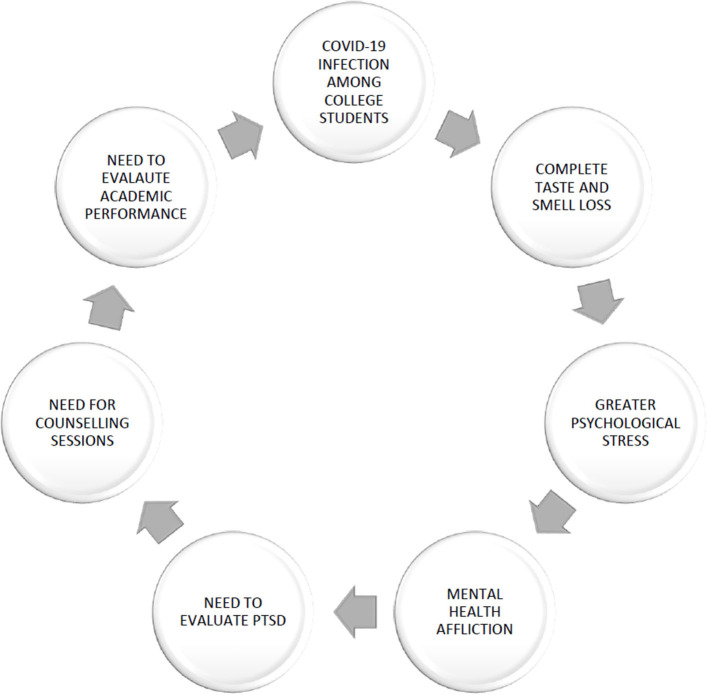

## Background

Since the outbreak of a novel coronavirus in December 2019 (which was first termed 2019-nCoV, then later SARS-CoV-2) a series of unprecedented events followed with the epicenter being in Wuhan, China (1). The global threat situation as categorized by WHO's for the pandemic was “very high” as it spreads very quickly among the healthy and diseased population with a higher mortality rate (2).

A series of worldwide lockdowns were imposed causing anxiety, depression, stress and sedentary behaviors among the healthy and diseased population (3). A study analyzing the psychological impacts in Saudi Arabia of corona virus disease (COVID-19) found that almost one fourth of the sampled population experienced moderate to severe symptoms (4). It is usually observed that the mental health evaluation of persons exposed to such natural tragedy experiences frequently report emotional health issues including post-traumatic stress disorders (PTSD), anxiety, fear and depression (5).

The norms of COVID-19 prevention were strictly enacted in the country, still due to high transmission rate present with the virus (2) it has affected the student population. Though death in younger population may be rare but, they may suffer from disruptive and even life altering perceptions (6). Another surprising discovery of COVID-19 is that the people affected with even milder strain of the virus may have long term complications of the disease (7). Long term complications may range from cardiovascular, neurological and musculoskeletal complications and the most commonly reported long term symptoms are fatigue and breathlessness (7).

As the transmission of the virus is high thereby isolation of the affected population is required. During the quarantine period usually the patients report psychological discomfort which could result in mental health problems in long run (8). It's been proven that COVID-19 survivors experience COVID-19 stigma, fear of eminent death, which may have long term impact (9).

Now there are also mounting evidences in the researches to suggest that stress can be caused by the disease process, anxiety of spreading the disease to others and COVID-19 humiliations can cause feeling of dejection among the survivors (9). Some researchers also suggested that there appears to be an increased risk of psychiatric consequences among the survivors (10). A recently published study found after 6 months of follow-up, lone survivors were primarily distressed by sleep difficulties, fatigue, depression and anxiety (11). The aforementioned studies clearly outline the development of psychological issues in COVID-19. Hence, from the previous researches it's quite obvious that it will be of vital importance to measure the psychological impact among young survivors post COVID-19 infection.

Originally the impact of event scale (IES) was developed for people experiencing post-traumatic stress disorders (12). It is the most commonly used scale for measurement of stress, anxiety and depressive symptoms after a traumatic event (13). Lately another version of the Impact of Events Scale-Revised (IES-R) is being inducted which is a 22-item self-reported scale for trauma related distress, validated in people with traffic injuries (12). The scale also have been adapted to measure the mental and psychological stress arising among the COVID-19 Survivors (14).

Commonly identified stressors among the college students include academic pressure, environmental pressure, social and interpersonal pressure (15). To elaborate, the student population have often encountered an increased psychological or mental health issues during the COVID-19 pandemic (15). The postulated reasons could be due to stress from long hours of online classes, exams, assignment submissions, no practical exposure, no recreational activities due to series of lockdown which may have further amplified the social stigma and psychological impacts from COVID-19. However, still we need to analyze the mental and psychological states of college students post COVID-19 recovery.

Hence the need of the present study is to understand the psychological and mental health of young student survivors in Saudi Arabia after getting infected with COVID-19 infection. So, the present objective of the research was to find the impact on psychological and mental health of the students' survivors at the university post SARS-CoV-2 infection.

## Methods

### Study Design and Sampling Technique

The study had university ethical clearance number, University Protocol Record ECM#2020-2501 and also registered at *ClinicalTrials.gov* with number # NCT04746443. The study was open for recruitment to the participants by December 2020. The study design of the experiment was cross-sectional, sampling technique being non-probability and sampling method being implied was convenience sampling.

### Setting and Study Procedures

The study was conducted in King Khalid University, Saudi Arabia among the college going students of physiotherapy, radiology and medicine program. A prior consent was taken before they participated in the online version if IES-R. In the current study the participants were given online instructions and education related to the questionnaire in study, the associated advantages and risks and the way in which data from the participants will be used for analyses. The participants were also assured that under all circumstances anonymity and confidentiality of the participants will be maintained.

The participants were asked to join the experimental survey only when the active phase of the disease or related symptoms was over, that was mostly after 20^th^ day, by then all study participants have recovered and resumed their diurnal activities and lifestyle. Before starting the survey, the participants were given sets of standardized instructions i.e., a brief how to fill the online survey. The IES-R is an easily self-administered 22-item questionnaire to evaluate the symptoms of posttraumatic stress disorder (PTSD) after any traumatic event. The scale consist of three parts mainly avoidance, intrusion, and hyper arousal. For each answers to the question it was rated using the score from zero to four, in the scale usually score of zero indicates “not at all” and score of four “extremely affected.” The overall score for IES-R was mainly classified as either into 0–23 which says the score is normal, a score of 24–32 predicts a mild affliction, score of 33–36 predicts moderate affliction, and a score >37 predicts severe psychological impact (4). Apart from the routine IES-R questions additional questions were also recorded like age, ethnicity, ability to smell, approximate date of the event and presence of any previous comorbidities.

The validity of IES-R is well-established to measure delayed onset of post-trauma frequencies. Such findings have already facilitated our understanding regarding evaluation and management in younger population in clinical and research scenario. Moreover, the scale also reports a high test–retest reliability among the affected population collected at 6-month interval (0.89–0.94) (16).

The inclusion criteria of the study were college going students who were tested positive on RT-PCR testing and exhibited symptoms in accordance with the guidelines laid down by ministry of Saudi Arabia. The exclusion criteria for the study was unwillingness to participate, negative RT-PCR testing, inability to understand the instructions for online survey, severe illness affecting cognitive and intellectual functions and hospitalization in ICU with mechanical ventilation. (https://www.moh.gov.sa/en/Ministry/MediaCenter/Publications/Pages/covid19.aspx).

### Data Analysis

The analysis was conducted using IBM SPSS Statistics for Windows, version 24. Each question in the survey were compulsory, that is, it was required to be filled before proceeding to the next questions. An analyses which mainly comprised of sociodemographic characteristics and symptom profile data (age and sense of smell) was showcased as descriptive statistics. The outcomes of these investigations were then showed as percentages and frequencies for categorical variables and means and standard deviations for continuous variables using the whole sample of *n* = 34. The results of which are presented in Table 1.

**Table 1:** Baseline characteristics of the participants in the study.

**Variables**	**Number (*n*) = 24**	**Mean**	**Standard deviation**
Age	24	21.42	1.21
Comorbidities	2 (Obesity)
IES-R (Total score)	24	21.25	18.1
IES-R (Intrusion)		20.38	10.13
IES-R (Avoidance)		23.88	6.56
IES-R (Hyperarousal)		26	8.46

The mental affliction of SARS-CoV-2 pandemic was evaluated utilizing the scores of the IES-R results described as mean and standard deviation. Further in the evaluation Bivariate analyses for correlation coefficients was used to a certain the strength of association amid discrete variables and scores the subscales of IES-R (Intrusion, Avoidance, and Hyperarousal) and the level of significance for the analysis was kept at *p* < 0.05. An Ordinal regression model was created using score rating (normal, mild, moderate, severe) on the IES-R as a dependent variable, and independent factors like sense of smell and covariate being age to know the degree of association of independent variable with dependent variable.

## Results

All participants were from Aseer province, Saudi Arabia. A total of 34 students were established to have been infected by SARS-CoV-2 as also confirmed by the university record. The students of physical therapy, radiology and medicine programs were contacted through the online link, to fill the IES-R English version. A second reminder was sent after a week if in case the participants didn't respond to the first reminder. Out of 34 only 24 male students responded to the online survey with an overall participation level of 71%. Apart from IES-R additional factors like age, ethnicity, existing co-morbidity and ability to taste and smell were also reported.

The participant's average age was [Mean ± Standard deviation (SD)] 21.63 ± 1.27 years. According to this classification scores of each participant were analyzed which is presented in Table 2. Out of 24 participants, 9 (38%) participants showed the symptoms of mild (*n* = 2) –severe (*n* = 7) psychological impacts.

**Table 2:** Itemized response of participants for each questions of IES-R.

**Questions**	**Not at all**	**A little bit**	**Moderately**	**Quite a bit**	**Extremely**
1. Any reminder brought back feelings about it	50%	33.3%	8.3%	-	8.4%
2. I had trouble staying asleep	45.7%	8.3%	8.3%	29.2%	8.4%
3. Other things kept making me think about it	54.1%	4.2%	29.2%	4.2%	8.3%
4. I felt irritable and angry	45.8%	16.7%	8.3%	16.7%	12.5%
5. I avoided letting myself get upset when I thought about it or was reminded of it	54.2%	4.2%	8.3%	8.3%	25%
6. I thought about it when I didn't mean to	58.3%	16.7 %	12.5%	12.5%	-
7. I felt as if it hadn't happened or wasn't real	66.7%	4.2%	12.5%	8.3%	8.3%
8. I stayed away from reminders of it	41.7%	33.3%	20.8%	4.2 %	-
9. Pictures about it popped into my mind	54.2%,	12.5%	8.3%	8.3%	16.7%
10. I was jumpy and easily startled	45.8%	37.5%	4.2%	8.3%	4.2%
11. I tried not to think about it	66.6%	12.5%	16.7%	-	4.2%
12. I was aware that I still had a lot of feelings about it, but I didn't deal with them	58.3%	12.5%	8.3%	12.5%	8.4%
13. My feelings about it were kind of numb	54.2%	12.5%	8.3%	16.7%	8.3%
14. I found myself acting or feeling like I was back at that time	62.5%	16.7%	12.5%	-	8.3%
15. I had trouble falling asleep	50%	-	20.8%	16.7%	12.5%
16. I had waves of strong feelings about it	58.3%	12.5%	20.8	4.2%	4.2%
17. I tried to remove it from my memory	54.1%	16.7 %	4.2%	-	25%
18. I had trouble concentrating	50%	-	25%	16.7%	8.3%
19. Reminders of it caused me to have physical reactions, such as sweating, trouble	66.6%	25%	4.2%	-	4.2%
breathing, nausea, or a pounding heart					
20. I had dreams about it	95.8%	-	4.2%	-	-
21. I felt watchful and on-guard	66.6%	4.2%	4.2%	8.3%	16.7%
22. I tried not to talk about it	66.6%	16.7%	4.2%	-	12.5%

### Analysis of Participant's Response IES-R (1–22)

The frequency analysis of responses for each 22 items in the IES-R scale was done to know the individual effect on each participant, it has been summarized in Table 2.

### Loss of Sense of Taste and Smell

Out of 24, 16 (66.66%) participants reported temporary, complete loss of sense of taste and smell whereas 1(4.17%) participant reported in temporary reduction in sense of taste and smell. The other 7 (29.17%) participants reported no loss of sense of smell during the COVID-19 infection process.

### Psychological Impact as Measured Among Participants Based on Score of IES-R

The result from IES-R were interpreted under the pathological range and classified as per the scores from previous study (4, 17–19), usually a score >23 was accepted to be in line with mental affliction. The scores were hence classified as 23 or more (mild) for persons who still do not have full blown symptoms of PTSD or at least some of the symptoms. Score of 33 (moderate) and above this represents the best cut off for a probable diagnosis of PTSD and a score of 37 (severe psychological impact) or more is high enough to suppress the immune system's functions. Seven participants out of 24 had a severe classification according to the scoring system, 2 participants had mild affection due to COVID-19, whereas 15 participants had normal scores.

### Correlation of Factors

Bivariate spearman correlation was done for factors like sense of taste and smell loss, age, IES-R total, intrusion, avoidance, hyperarousal scores. On correlation of factors like total IES-R score and sense of smell was significant with moderately negative association (−0.58^*^, *p* = 0.003). Whereas, intrusion (0.11, *p* = 0.79), avoidance (−0.23, *p* = 0.59), hyperarousal (−0.11, *p* = 0.84) and age (−0.31, *p* = 0.14) aspect of IES-R was found to have very weak to weak correlation respectively.

### Ordinal Regression

A regression model was created using score rating (normal, mild, moderate, severe) on the IES-R as a dependent variable, independent factor as sense of smell and covariate being age. The model of fitting information was significant at *p* = 0.02 (which implies we reject the null hypothesis i.e., there was a significant difference between the baseline model (dependent variables) to final model (independent variables), goodness of fit the Pearson value was > 0.05 signifying a good statistical model between observed data and the fitted data in the model. A variance of [Pseudo r^2^, (Nagelkerke)] 44.2% was found, which tells us the proportions of variance as explained by the independent variable on the dependent variable in the regression model. The parameters estimate for age had a value of −0.45 which was non-significant and for *complete* loss of sense of smell the estimate was −19.3 which was significant, for *reduction* in sense of smell the estimate was −0.356 with a non-significant. Thus, implying a significant difference between the IES-R score ratings of participants with complete loss of sense of smell as compared to reduced or normal sense of smell. In addition, the test of parallel lines was non-significant.

## Discussion

This is the first study with the primary aim to address the psychological and mental impacts among young student's survivors post COVID-19 infections.

In the present study post COVID-19 infection, 38% of the population showed the symptoms of mild to severe psychological impacts. A study on anxiety and depression among COVID-19 survivors found that infection triggered turbulences in the immune system may start a vicious inflammatory cycle which may lead to psychiatric issues or worsening of symptoms among the survivors (20). It now evident that the psychiatric sequel to the infection may be caused by the virus or by psychological stress factors like social isolation, social stigma, rejection and living in limbo due to the risk of fatalities (9, 20). The immune reaction in the body to the coronavirus may pave way for local and systemic inflammatory process (20, 21) leading to insomnia and further aggravation of the impending psychiatric responses.

Almost 29% of the population abstained from answering the present surveys. A postulated reason could be social stigma. Social stigma in COVID-19 may be defined as a state in which people with potential source of infection may be excluded from daily social practices posing a risk to the active social living in the society (22). The magnitude of the problem may further be amplified with perceived racial, religion, tribe or caste discriminations (23).

Another interesting finding of the current study was loss of *LOTAS* among the participants which was found to be approximately 67%. In addition, *LOTAS* was found to be associated with the severity of the IES-R. Though the exact mechanism highlighting it isn't evident but this problem has been primarily postulated to central nervous system dysfunction (24) raising the wide possibilities for variety of emotional disturbances among the population. The present study is in line with studies which outline olfactory dysfunction to be strongly associated with psychological sequelae. Though the study didn't report any mortality among the participants, which is also commonly associated with loss of sense of smell and taste (24) among the afflicted population.

The diagnostic ability of IES-R which compared war survivors from two large populations found the self-reported questionnaire to be clinically useful in screening patients of post-traumatic stress disorders (PTSD) (25). The authors also found that cut-off scores from 22, 30, 33, and 44, respectively are linked with good to excellent sensitivity (25). Even in the present study it's evident that the college going students did suffer from traumatic episodes post COVID-19 recovery.

The biological links between COVID-19 and mental health among survivors certainly cannot be ignored. The immune-inflammatory modulation during COVID-19 and recovery may pave its way for mental illness (26). There is a need for development of student counseling strategies to control and minimize the long-term mental impacts among them. To add further, there is also a need to establish an online mobile helpline support for younger survivors', increase awareness regarding coping up strategies and assessment of interpersonal problems (27) will play a foremost role in promoting wellbeing. A metanalysis has also concluded that the mental health interventions can play a positive constructive role for promoting metal wellbeing among the survivors of COVID-19 (27).

In the present study 2 obese students were also afflicted with COVID-19. The prevalence rate of metabolic syndrome in Saudi Arabia is already found to be near about 39.8% according to the data obtained from National Cholesterol Education Program and Adult Treatment Panel III (NCEP ATP III) (28), further studies also suggest that prevalence of overweight, obesity, hypertension and diabetes are also high in the region (29). These factors will accelerate the unfavorable outcomes among the survivors. A study outlined that obesity and pre-existing co-morbidities may increase the admissions to intensive care unit, lung damage and mortality among those affected by the SARS-CoV-2 virus (30). To add further, there is still ambiguity in data how the presence of comorbidities may affect the COVID-19 disease process in the younger population with economic, geographic and biological differences, hence there is better need for guidelines for rehabilitation and decision making process for children's, adolescent and young adults affected (31).

**Figure 1:**
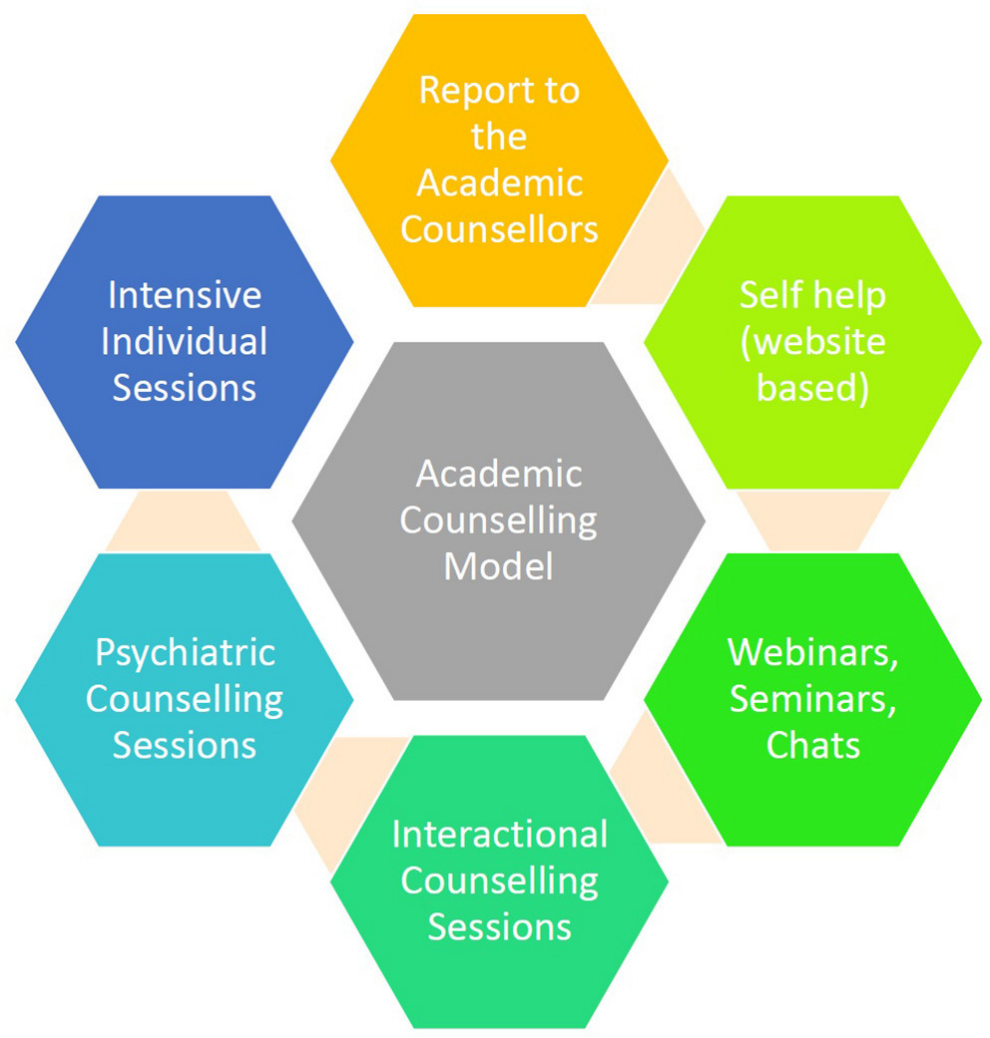
Outlines the Counseling model to be used in the academic institutions if an individual gets afflicted with COVID-19.

The current need is to develop a model for younger population afflicted by COVID-19 (Figure 1) in academic institutions. There is strong evidence that exercises may not only modulate cardiovascular risk factors but can also promote a feeling of wellbeing among those affected with COVID-19. It is also vital to encourage students post COVID-19 recovery to participate in mild to moderate activity as a strategy for self-management of anxiety and mood problems (32) among those affected. It is also reported that sports person who are physically active pose a lesser risk of transmission (33) a postulated reason could be physical activity modulate the immune defense mechanism (34) and a recent guidelines also emphasizes the need for staying active due to its multiple optimistic effects with the requirement to bring together clinical services together to address the current issue (35).

### Study Strength and Limitations

The study foremost strength is to identify the existence of psychological and mental health issue post COVID-19 episode among the survivors at the university. The severity of the IES-R score may depend on the complete loss of taste and sense of smell. The study also emphasizes the need for the development of an academic counseling model to be used at the college and university level to address the issue as it may affect the academic performances of the students. The notable limitations were a smaller sample size, as the incidence rate was lesser during the study period. Additionally, other factors were not included such as socio-demographic factors, gender, BMI, Hospitalization/ home isolation, treatment, prior physical activity levels which are known independent variables and could have influenced the mental health. A part from that all the participants were male who were included in the study and didn't had any previous history of mental or emotional illnesses.

## Conclusion

The study clearly outlines the presence of psychological sequels post recovery of COVID-19 infection among the young college going population. The students with complete loss of taste and smell may be more adversely affected than those who didn't experience the complete loss of taste and smell. It is also quite evident that there is a need for academic counseling models for the students affected.

## Data Availability Statement

The raw data supporting the conclusions of this article will be made available by the authors, without undue reservation.

## Ethics Statement

The studies involving human participants were reviewed and approved by University Protocol Record ECM#2020-2501. The patients/participants provided their written informed consent to participate in this study.

## Author Contributions

SD: study conception and design. SD, AMu, VK, and RR: data collection. SD, AB, and MA: analysis and interpretation of results. FC, SG, and AMo: data cleaning. SD, AB, and FA: draft manuscript preparation. All authors reviewed the results and approved the final version of the manuscript.

## Funding

This work was funded by King Khalid University, RGP.2/134/43.

## Conflict of Interest

The authors declare that the research was conducted in the absence of any commercial or financial relationships that could be construed as a potential conflict of interest.

## Publisher's Note

All claims expressed in this article are solely those of the authors and do not necessarily represent those of their affiliated organizations, or those of the publisher, the editors and the reviewers. Any product that may be evaluated in this article, or claim that may be made by its manufacturer, is not guaranteed or endorsed by the publisher.

## Abbreviations

SARS-CoV-2: severe acute respiratory syndrome coronavirus 2
COVID-19: Coronavirus disease of 2019
IES-R: Impact of event scale (IES)–Revised
NCEP ATP III: National Cholesterol Education Program and Adult Treatment Panel III
PTSD: Post-traumatic stress disorders
LOTAS: Loss of sense of taste and smell
WHO: World Health Organization
RT-PCR: Reverse Transcription–Polymerase Chain Reaction.
